# Electrochemical sensor for the detection of adrenaline at poly(crystal violet) modified electrode: optimization and voltammetric studies

**DOI:** 10.1016/j.heliyon.2022.e10835

**Published:** 2022-10-04

**Authors:** Saheed E. Elugoke, Omolola E. Fayemi, Abolanle S. Adekunle, El-Sayed M. Sherif, Eno E. Ebenso

**Affiliations:** a Department of Chemistry, School of Physical and Chemical Sciences, Faculty of Natural and Agricultural Sciences, North-West University (Mafikeng Campus), Private Bag X2046, Mmabatho 2735, South Africa; b Material Science Innovation and Modelling (MaSIM) Research Focus Area, Faculty of Natural and Agricultural Sciences, North-West University (Mafikeng Campus), Private Bag X2046, Mmabatho 2735, South Africa; c Department of Chemistry, Obafemi Awolowo University, Ile-Ife 220005, Nigeria; d Research Chair for Tribology, Surface, and Interface Sciences (TSIS), Department of Physics and Astronomy, College of Science, King Saud University, P.O. Box 2455, Riyadh 11451, Saudi Arabia; e Center of Excellence for Research in Engineering Materials (CEREM), King Saud University, P.O. Box 800, Al-Riyadh 11421, Saudi Arabia; f Centre for Material Science, College of Science, Engineering and Technology, University of South Africa, Johannesburg 1709, South Africa

**Keywords:** Electropolymerization, Adrenaline, Electrochemical impedance spectroscopy, Crystal violet, Square wave voltammetry

## Abstract

Herein, we report the electropolymerization of crystal violet (CRV) on a bare glassy carbon electrode (GCE) for the detection of adrenaline (AD). Electropolymerization parameters such as electrolyte pH, scan rate and monomer concentrations were optimized using cyclic voltammetry (CV) and electrochemical impedance spectroscopy (EIS). The characterization of CRV and poly(crystal violet) (PCV) was done using FT-IR, UV-visible spectroscopy and EIS. More importantly, the charge transfer resistance (R_ct_) and other EIS data recorded from the EIS of various forms of the poly(crystal violet) (PCV) modified glassy carbon electrode (GCE) in AD were used for identifying the best PCV modified electrode. Subsequent application of the electrode prepared at optimum conditions (PGCE) for AD detection using the square wave voltammetry (SWV) gave a limit of detection (LOD) of 2.86 μM over a linear range of 10.3–102.7 μM. This sensor also showed considerable stability, good AD recovery from the real sample (98.9%), and excellent reproducibility, making it a suitable analytical tool for AD detection at the micromolar level.

## Graphical abstract

**Figure:**
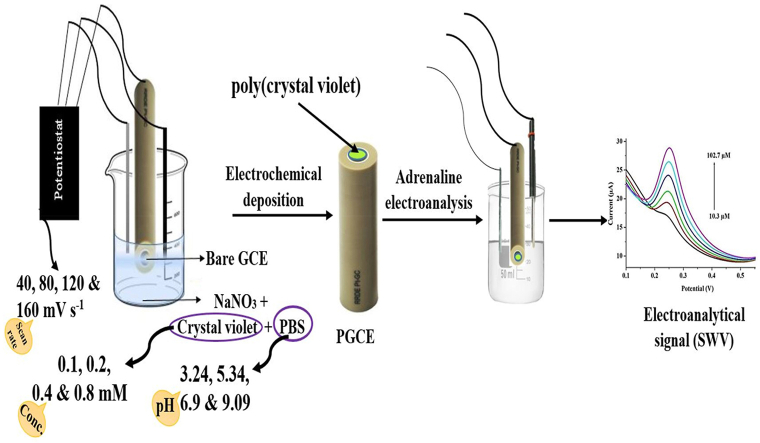

## 1 Introduction

In the last few years, thin polymer films have gained tremendous attention as modifiers for electrodes targeted toward detecting biomolecules and a wide range of other chemical substances. The ease of formation of these films, selectivity for the chemical species of interest, and the total cost of fabrication of the modified electrode are a few reasons for this trend. Dye and amino acid molecules have enjoyed special patronage in this recent streak of facile sensor fabrication [1, 2, 3, 4]. Specifically, the detection of dihydroxybenzenes down to very low concentrations has been achieved through the simple modification of conventional glassy carbon, platinum, carbon paste, and gold electrodes with the polymeric form of these organic molecules [1, 2, 5]. For instance, the carbon paste electrode has been modified with poly(naphthol green B) for hydroquinone and catechol detection. In the same vein, poly(murexide) and the polymeric form of some other organic molecules have been utilized to modify carbon paste electrode to obtain a sensing platform for these dihydroxybenzenes [6, 7]. Also, polymerized dyes and amino acid modified electrodes have been applied as electrochemical sensors for neurotransmitters and other biomolecules [8, 9, 10]. Compared to the amount of literature available for electrochemical sensing application of these classes of materials, fewer reports on neurotransmitter detection at these simple electrodes abound.

Crystal violet (CRV) is one of the most prominent organic dyes with a high degree of aromaticity owing to the intrinsic three benzene rings. As a result, the polymerization of CRV gives a polymer with intact π-bonds that makes the formation of a π-π interaction with a molecule with such π-bonds stronger, thus enhancing the electrocatalytic activity of the polymer towards such analyte. Poly(crystal violet) (PCV) also has excellent catalytic activity towards the reduction of cations [11]. Consequently, its application as the modifier of electrodes for detecting metals in aqueous media had been attempted with huge success. In a certain instance, PCV has served as a catalytic layer of a graphene-modified glassy carbon electrode (GCE) to obtain a much better current response through the synergy between PCV and the underlying graphene [11]. Similarly, PCV layers have been electropolymerized on MWCNTs modified GCE to detect luteolin (a flavonoid) [12]. In the current study, we investigated the electrocatalytic activity of a simple PCV-modified GCE (fabricated via the electropolymerization of CRV on GCE) towards adrenaline oxidation. To the best of our knowledge, there is a paucity of literature on the electrocatalytic detection of AD using PCV-modified electrodes. Thus, this work was inspired by the catalytic activity of poly(crystal violet) modified electrodes towards catechol, luteolin and hydroquinone oxidation [5, 12, 13]. It is expected that the large surface area of PCV and the π-π interaction between these molecules and PVC [13] which enhanced their electrocatalytic oxidation would be replicated with adrenaline at a PVC-modified GCE (PGCE). In addition, the ease of fabricating PGCE via the electrodeposition approach which requires limited human efforts gives the proposed sensor an advantage over some existing AD sensors fabricated through the more laborious drop-casting technique [14, 15, 16] and some other modification techniques [17, 18, 19].

Adrenaline (AD), otherwise called epinephrine is a neurotransmitter popularly referred to as a ‘flight or fight’ neurotransmitter [20]. Like every other endogenous chemical substance with the ability to provoke physiological changes in the human system, the regulation of adrenaline in the human system is essential for healthy living. Noteworthy, AD imbalance in the human system has been reported to be a possible cause of life-threatening ailments such as Schizophrenia, anxiety disorder, Parkinson’s disease, among others [21, 22, 23]. At conventional electrodes, the detection of AD in the presence of other biomolecules such as ascorbic acid (AA) and uric acid (UA) is very difficult because these analytes often oxidize at very close potentials in the presence of these unmodified electrodes. Beyond the actualized better resolution of the oxidation peaks of AD and the interfering AA at the PCV-modified GCE fabricated in this study, we carried out a thorough electrochemical optimization of the CRV polymerization parameters for the first time. The electrocatalytic activity of the simple PCV modified GCE (PGCE) in the presence of AA was also investigated. To the best of our knowledge, this is the first attempt to optimize PCV films with a combination of CV and EIS. This work is equally the first time a PCV-modified GCE will be applied for the electrochemical detection of AD or any other neurotransmitter.

## 2 Experimental

### 2.1 Materials

Sodium nitrate (NaNO_3_) (98.5 %), crystal violet (>85 %), sodium dihydrogen phosphate (NaH_2_PO_4_) (99 %), disodium hydrogen phosphate (Na_2_HPO_4_) (99 %), epinephrine hydrochloride, adrenaline injection and ascorbic acid (99.5 %) were supplied by Merck chemicals, Wadeville, Gauteng, South Africa. Phosphate buffer saline (0.1 M, pH 6.9 & 7.0) (PBS) was prepared using the appropriate amount of NaH_2_PO_4_ and Na_2_HPO_4_ in distilled water. 0.1 M PBS of pH 6.9 and 7.0 were used for CRV polymerization and AD electroanalysis, respectively. All reagents were of analytical grade.

#### 2.1.1 Apparatus

The FT-IR spectroscopy was carried out using Opus Alpha-P spectrophotometer over a wavenumber of 400–4000 cm^−1^. Spectroquant 600 spectrophotometer (Merck, Germany) was used for UV-visible analysis. All electrochemical studies were done at a PGSAT Autolab workstation running on Nova 1.1 software. The three-electrode system attached to the workstation comprises a platinum counter electrode, Ag/AgCl (saturated KCl) reference electrode, and the PGCE as the working electrode.

### 2.2 Methods

#### 2.2.1 Modification of bare GCE

The electropolymerization of CRV on the bare GCE was done using a method adopted by Chen et al. with slight modification [11]. Briefly, bare GCE was cleaned using an alumina slurry on a cleaning pad and subsequently ultrasonicated, first in methanol and then in distilled water before drying in the oven at 50 °C. The clean bare GCE was immersed into a solution containing 10.00 mL of 0.1 M PBS (pH 6.9), 4.00 mL of 0.2 mM CRV and 6.00 mL of 0.05 M NaNO_3_. Then, the electropolymerization was done by running 12 cyclic voltammetry (CV) scans over a potential window of −1.2 to +1.8 V at a scan rate of 80 mV s^-1^. The surface of the modified electrode (PGCE) was rinsed with a copious amount of water to remove unpolymerized CRV monomers. The modified electrode was dried in an oven at 50 °C to reveal a golden yellow coat over the surface of the electrode. The optimization of the polymerization parameters was done by also using 0.1 mM, 0.4 mM and 0.8 mM CRV and by varying the pH of the buffer (at the optimum CRV content). The scan rate applied for the electropolymerization was equally optimized over a scan rate range of 40–160 mV s^-1^.

#### 2.2.2 Preparation of real sample

The adrenaline injection for real sample analysis was prepared by diluting 0.05 mL of the ampoule with 100 mL of PBS (pH 7). This solution was further diluted to obtain 1000 times dilution of the injection.

## 3 Results and discussion

### 3.1 Characterization of CRV and PCV film

The electropolymerization of the monomer on the bare GCE was optimized by varying the pH, scan rate and monomer (CRV) quantity applied for the electropolymerization. The current response obtained at the modified electrode in a solution of 0.4 mM AD in 0.1 M PBS (pH 7.01) using CV and electrochemical impedance spectroscopy (EIS) spectra obtained in the same solution was used for monitoring the optimization processes.

**Figure 1:**
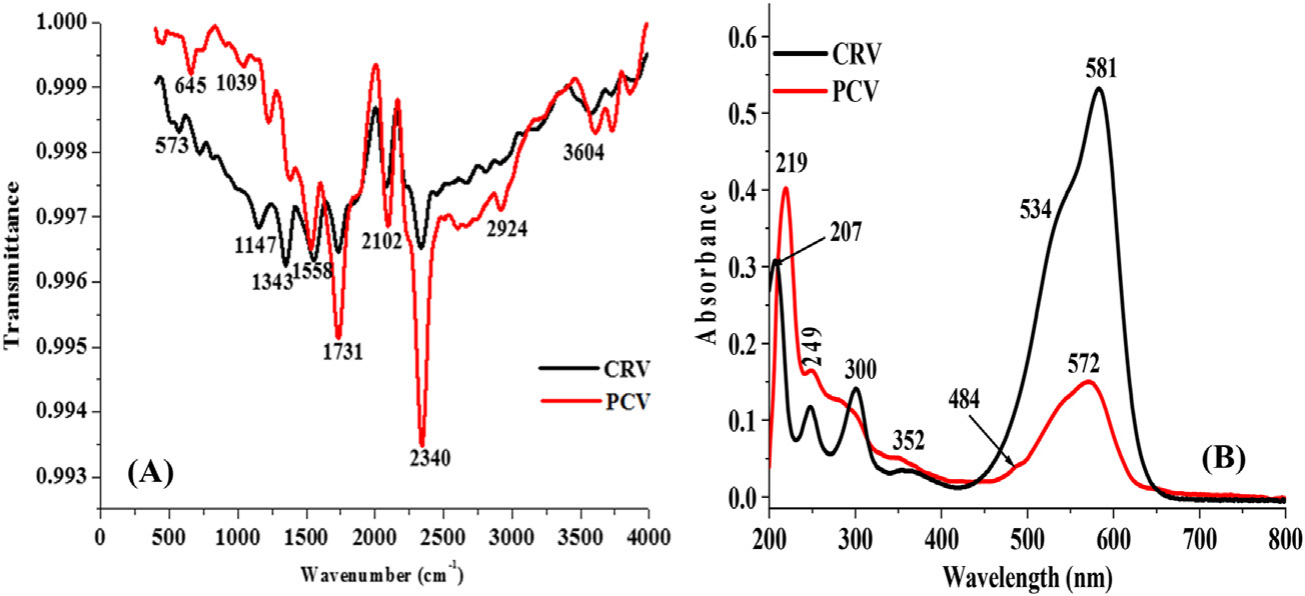
(A) FT-IR spectra and (B) UV-visible spectra of CRV and PCV.

**Scheme 1:**
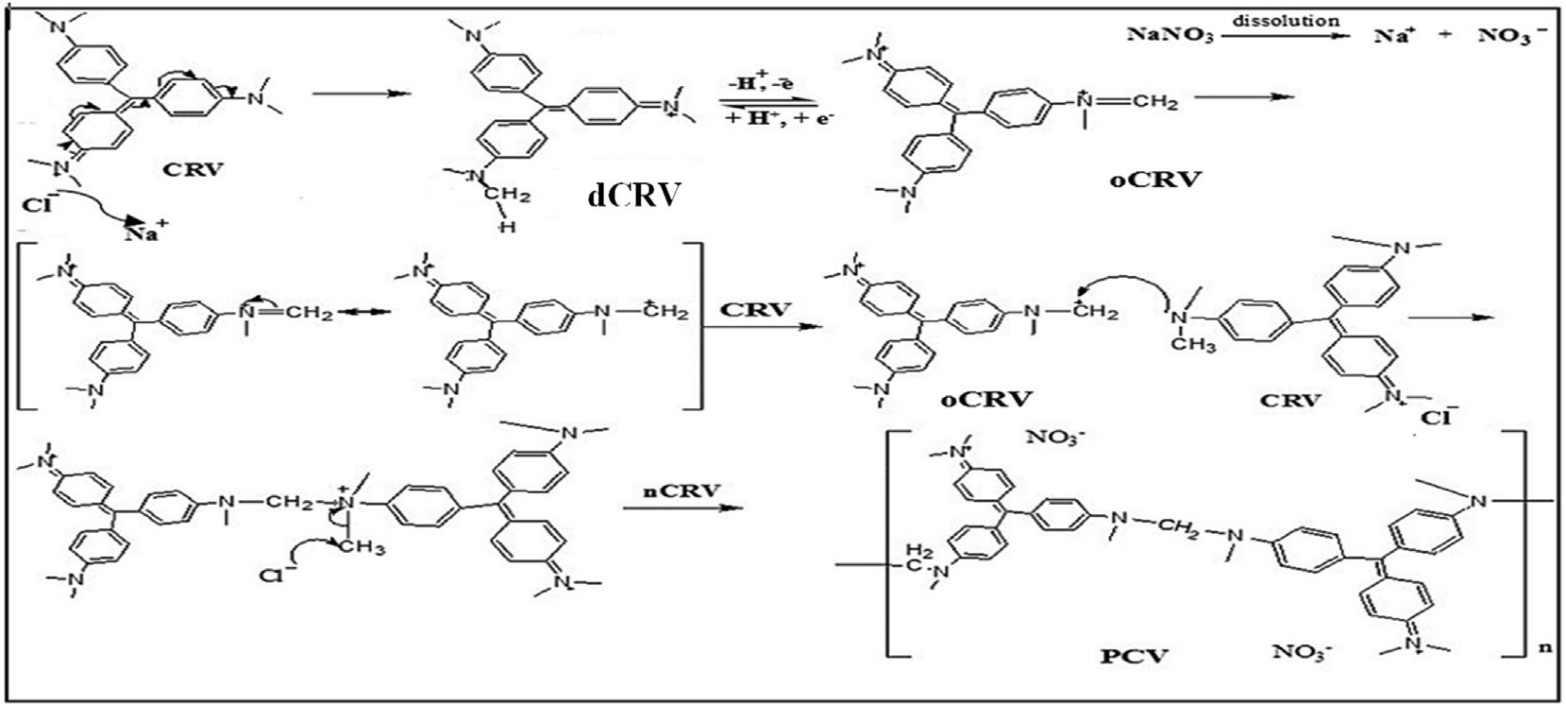
Mechanism of CRV polymerization.

The FT-IR spectra of CRV and PCV were obtained over a wavenumber range of 400–4000 cm^−1^ (Figure 1A). The absorption bands at 995, 1343, and 1558 cm^−1^ appeared on the CRV spectrum due to C=C bending vibration of a disubstituted benzene ring, C–N stretching of aromatic amine, and the C=C stretching vibration of extensively conjugated C=C bond, respectively (Scheme 1). Also, the absorption bands at 2102 and 2924 cm^−1^ were found on CRV spectrum due to the presence of aromatic C–H bending vibration and the aliphatic C–H stretch of the methyl groups, respectively. The FT-IR spectrum of CRV looks very much like that of PCV in several ways but it is slightly different. This is because the expected repeating unit of PCV would likely contain similar functionalities as that of CRV. However, there was an emergence of a peak at 1039 cm^−1^ (on the PCV spectrum) which could hardly be found on the spectrum of CRV, signalling the possible emergence of a new C–N bond after the polymerization of CRV. UV-visible spectroscopy was also applied for the analysis of CRV and PCV (Figure 1B). The UV-visible spectrum of CRV shows peaks at 249 and 581 nm due to the presence of chromophores and the three benzene rings. A similar UV-visible spectrum has been reported for CRV [24]. The CRV spectrum also revealed peaks at 207, 300, 352 and 534 nm. On the other hand, the UV-visible spectrum of PCV shows peaks at 219, 249, 300, 352, 484 and 574 nm. The peak at 484 nm on the PCV spectrum, which is a shift of the peak on the CRV spectrum (at 534 nm) to a lower wavelength indicates that a material different from CRV has been prepared after electropolymerization. After electropolymerization, the surface of the modified electrode gave a sparkling golden yellow colour which is different from the initial purple colour of the CRV. The fact that the peak at 534 nm on CRV spectrum is absent on the PCV spectrum also confirms the formation of the polymer.

### 3.2 Mechanism of CRV polymerization

**Figure 2:**
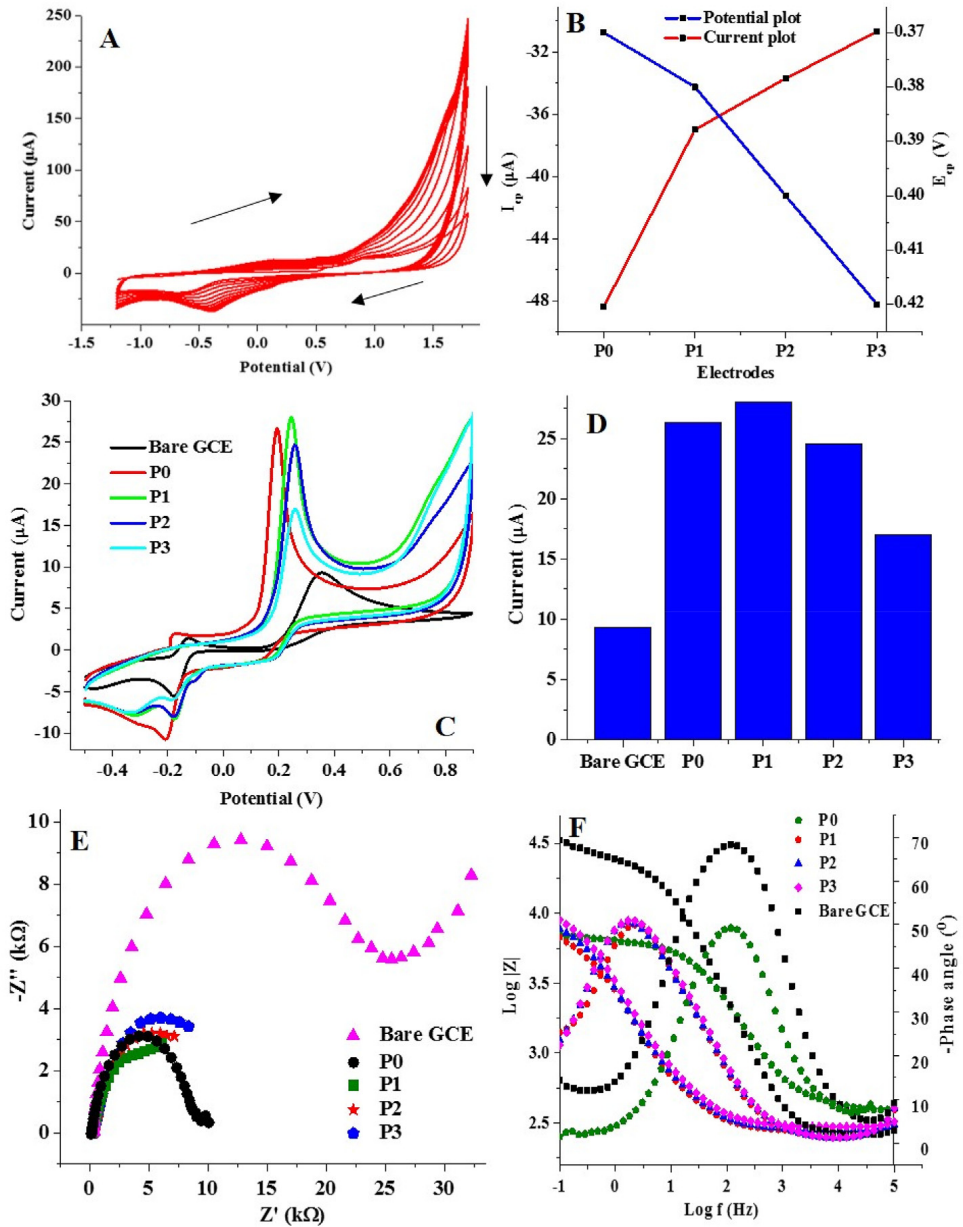
(A) Cyclic voltammogram of CRV polymerization with 0.2 mM CRV for P1 preparation (scan rate, 80 mV s^-1^), (B) Chart showing the current response of bare and modified GCE to various concentrations of CRV, (C) Cyclic voltammogram showing the current response of electrodes to AD oxidation at a scan rate of 25 mV s^−1^ in PBS of pH 7, and (D) Chart showing the current response of the electrodes to AD oxidation. (E) Nyquist, and (F) Bode and phase angle diagram of electrodes in 0.4 mM AD at a pH of 7.

The proposed mechanism of CRV polymerization (Scheme 1) shows a possible initiation of the polymerization process by the solvation of sodium nitrate (NaNO_3_), leading to the formation of the sodium and nitrate ions. The subsequent rearrangement of CRV after the loss of the counter chloride ion to Na^+^ gave rise to an oxidized form of CRV (oCRV) which subsequently helped in the propagation of the polymer chain with CRV molecules. The emergence of redox peaks at +0.23 V and −0.3 V (Figure 2A) can be ascribed to the formation of oCRV and the reduction of oCRV to the dechlorinated form of CRV (dCRV), respectively. Continuous CV scans made available oCRV which is essential for chain propagation. Chain termination took place by the combination of ions to form stable compounds such as chloromethane and some other inorganic moieties. Noteworthy, the surface of the bare GCE revealed a sparkling golden yellow PCV film after the electropolymerization process.

### 3.3 Electrochemical characterization of modified electrodes

#### 3.3.1 Effect of CRV concentrations

Using CRV concentrations of 0.1 mM, 0.2 mM, 0.4 mM, and 0.8 mM and keeping constant other parameters (as stated in the previous section), the resultant PGCE tagged P0, P1, P2 and P3, respectively, gave modified GCE with better electrocatalytic activity towards AD compared to the bare GCE (Figure 2A). The maximum CRV oxidation current response was recorded when 0.2 mM CRV was used to prepare the PCV film compared to PCV coatings with 0.1 mM, 0.4 mM and 0.8 mM CRV respectively (Figure 2B). Noteworthy, the current response recorded with 0.1 mM is very close to that of PCV prepared with 0.2 mM. This indicates that fewer CRV molecules in the electrolyte mixture (as was the case with P0 and P1) have easier and faster mobility towards the bare electrode and consequently oxidize at the electrode surface faster than the other CRV concentrations (0.4 and 0.8 mM). This can as well be attributed to a possible decline in the intermolecular interaction (van Der Waal force) between the individual CRV molecules when fewer quantities are used. A similar trend was observed when 1 mM CRV was used to modify graphene-supported GCE [11]. Modified electrodes P0, P1, P2, and P3 gave peak AD oxidation currents of 26.7, 28.04, 24.57, and 17. 04 μA, respectively (Figure 2C & D). These current responses are much higher than those recorded at the bare GCE (9.32 μA). Specifically, the oxidation peak current at P1 is about three times that of the bare GCE. The CV for the electropolymerization of the CRV concentrations in P1 revealed a gradual decrease in the oxidation current after the first three cycles, which appeared very stable up to the third scan (Figure 2A). In P0, P2 and P3, the current drop was evident throughout the 12 scans (Figure S1). This confirms that PCV formation was achieved after the first three scans at P1 while the formation of the PCV films at P0, P2 and P3 required further CV scans. It is also important to note that the CRV cathodic peak current increases with an increase in CRV content (Figure 2B). The Nyquist plot of the EIS spectra of each of the electrodes also revealed that the lowest R_ct_ was obtained at P1 (Figure 2E, Table 1), thus confirming the results obtained using CV. These findings were corroborated by the Bode plot of the EIS spectra going by the value of the impedance modulus at the lowest frequency (|Z| _at 0.1 Hz_) (Figure 2 F). Interestingly, the lowest value of |Z| _at 0.1 Hz_ (6.73 kΩ) was also recorded at P1. These results suggest that the optimum amount of CRV required for the best oxidation of AD is 0.2 mM. In addition, the EIS data revealed that the best conductivity could be achieved with low CRV contents. This could be ascribed to the fact that 0.1 mM and 0.2 mM CRV probably gave uniform and homogeneous bare GCE coating with the right thickness such that AD molecules could better oxidize at P0 and P1. In addition, the π-π interaction between P1 and AD was probably more effective at these electrodes because the electron transfer kinetics across a membrane with lower thickness and in proximity to the analyte might be faster. This observation was supported by the higher CRV current response and lower peak potential recorded at P0 and P1. Noteworthy, low potential implies faster reaction kinetics.

**Table 1:** EIS data of bare and modified electrodes (at various CRV concentrations) in AD.

Electrode	R_ct_ (kΩ)	R_s_ (kΩ)	Y_o_ (μΩ^−1^s^n^)	n	|Z| _at 0.1 Hz_ (kΩ)	*X*^2^
Bare GCE	22.40	0.26	1.30	0.893	33.26	0.0764
P0	9.83	0.31	1.89	0.808	9.96	0.1262
P1	8.45	0.28	87.8	0.734	6.73	0.0822
P2	10.40	0.29	86.1	0.729	18.17	0.0951
P3	11.80	0.30	74.2	0.735	8.98	0.0887

The relationship between PCV thickness at P1 relative to that of other modified electrodes could be inferred from the magnitude of the constant phase element CPE (Y_o_). Assuming that the polymer film on the GCE surface is a dielectric material, the value of Y_o_ gives an estimate of the pseudocapacitance of this polymeric coating (PCV). As seen in Table 1, the highest value of Y_o_ was obtained from P1. Noteworthy, the higher the thickness of the dielectric, the lower the resultant capacitance [25]. The exceptionally low Y_o_ value for P0 compared to other modified electrodes suggests that P0 possess a morphologically different material whose surface electrically mimics that of bare GCE due to the low CRV concentration applied for its fabrication. It is also important to note that the values of the exponent of the constant phase element (n) at P1, P2 and P3 are further away from unity than that of other electrodes. This is because the PCV coating on the surface of each of these electrodes gave rise to a modified electrode with greater deviation from an ideal capacitance than P0 and the bare GCE. More importantly, the close R_ct_ values at P0 and P1 explains why they have a similar current response to AD (Figure 2C).

**Figure 3:**
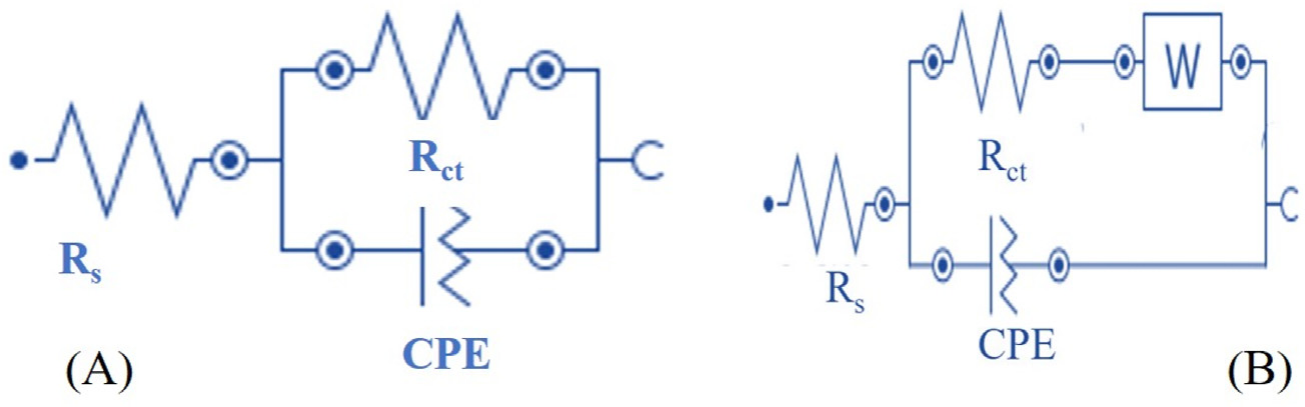
Equivalent electrochemical circuit for (A) PCV modified electrodes and (B) bare GCE.

The significance of the phase angle diagram and n is better explained when the best electrode (P1) was fabricated using various scan rates and then applied for the electrocatalysis of AD oxidation, as would be discussed later. The bare GCE showed a Warburg impedance (W) that was included in fitting the EIS data to get a better fit than the fit obtained without W. However, the equivalent circuit for the EIS data for the other electrodes was simulated without W, thus explaining why two different equivalent circuits have been used for modelling the EIS data (Figure 3A & B). This is the case with all the optimization done with EIS.

#### 3.3.2 Effect of pH on PCV formation

**Figure 4:**
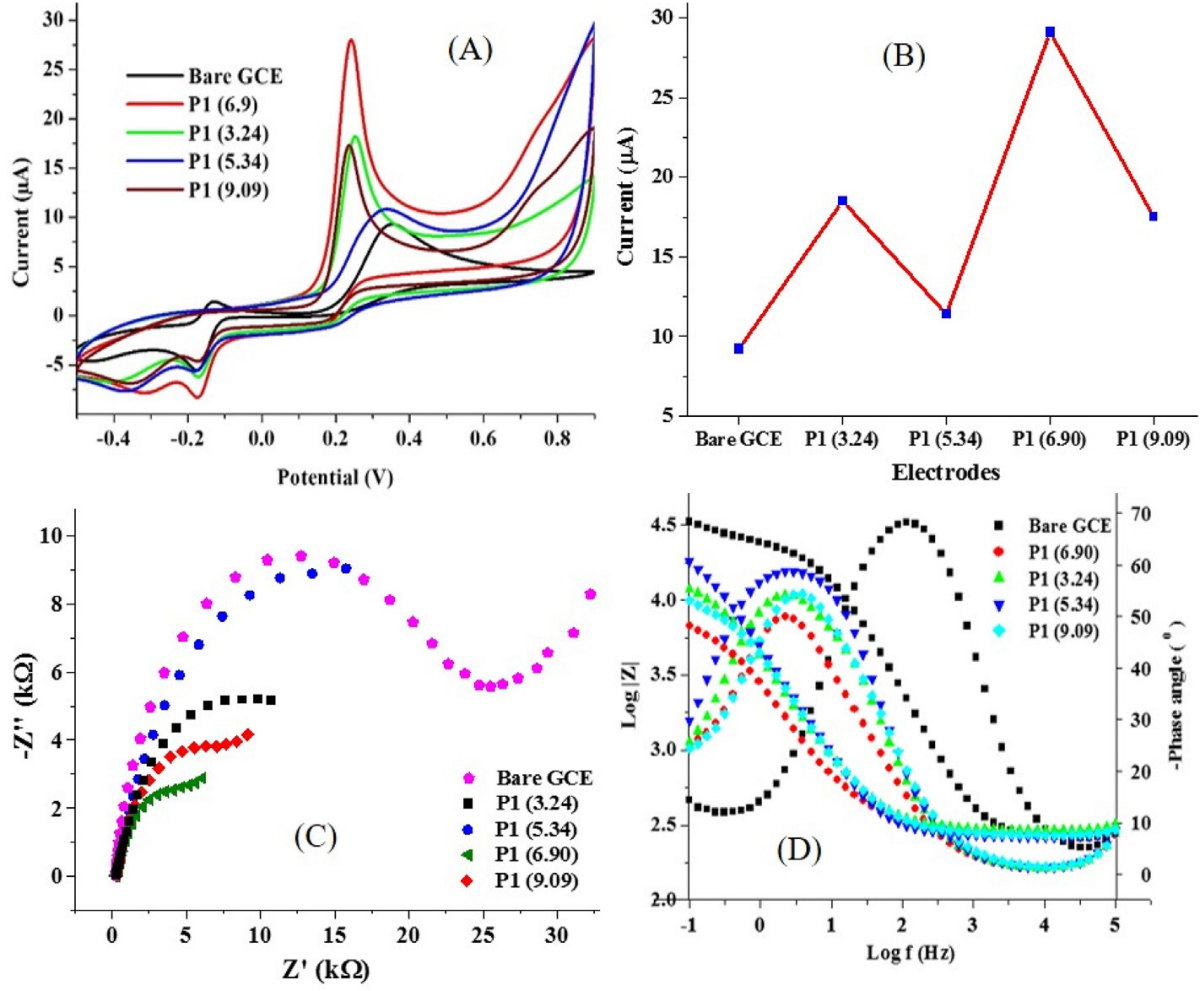
(A) Cyclic voltammogram of AD detection at bare GCE modified with PCV at various electrolyte pH and (B) Corresponding current response chart of (A). (C) Nyquist, and (D) Bode and phase angle diagram of the electrodes in 0.4 mM AD at a pH of 7 and scan rate of 25 mV s^−1^.

The optimization of the pH of the buffer used for CRV polymerization was done by varying the pH of PBS from 3 to 9 using the polymerization conditions in P1 to obtain four different electrodes tagged P1 (3.24), P1 (5.34), P1 (6.90) and P1 (9.09), respectively. Noteworthy, P1 (3.24), P1 (5.34), P1 (6.90) and P1 (9.09) represent electrode P1 fabricated after polymerization of CRV at pH 3.24, 5.34, 6.9 and 9.09, respectively. Figure S2 depicts the cyclic voltammograms recorded after the electrodeposition of PCV at all pH. Using the same mode of assessment as the CRV content in section 3.3.1, the highest oxidation current was obtained using P1 (6.9) (Figure 4A & B). With P1 (5.34), a potential shift (positive) from the AD oxidation potential witnessed with other electrodes was observed. This could be because P1 (5.34) possesses a morphology and surface area that raises the electrochemical potential between AD and the PCV film. A similar morphology-dependent electrocatalytic activity has been reported [26]. The EIS plots (Nyquist and Bode plot) also confirmed that the lowest R_ct_ (8.45 kΩ) and |Z| _at 0.1 Hz_ (6.73 kΩ) were recorded at a pH of 6.9 (Figure 4C & D). In addition, the EIS data in Table 2 suggest that the pH of the buffer impacts the conductivity of the resultant PGCE due to the possible effect of pH on the dispersion of CRV molecules in solution prior to polymerization. The Bode plot and the phase angle diagram confirmed this possibility considering the resistive behaviour of P1 (6.9) over a slightly wider frequency range than the other electrodes (as seen in the Bode plot) and the significantly lower phase angle relative to other electrodes (Figure 4D). It is important to note that the deviation of a material from an ideal capacitor can be estimated from the difference between the phase angle obtained with the material and a phase angle of -90 °. The greatest deviation from this ideal phase angle for a capacitor was obtained with P1 (6.9). The superior electronic conductivity of P1 (6.9) thus established further validates its superior current response to AD oxidation. Noteworthy, the bare GCE showed a Warburg impedance (W) which suggests possible diffusion of AD molecules towards the bare GCE [27, 28, 29], but the tail of the Nyquist plot of P1 (6.9) and P1 (9.09), which suggest a similar occurrence is somewhat misleading. This is because the inclusion of W in the simulation of the EIS data of these electrodes (P1 (6.9) and P1 (9.09)), gave a terrible fit, hence the adherence to the three-element equivalent circuit for all the modified electrodes and the four-element circuit for the bare GCE (Figure 3A & B).

**Table 2:** EIS parameter of bare GCE and GCE modified at various pH.

Electrode	R_ct_ (kΩ)	R_s_ (kΩ)	Y_o_ (μΩ^−1^s^n^)	n	|Z| _at 0.1 Hz_ (kΩ)	*X*^2^
Bare GCE	22.40	0.26	1.30	0.893	33.26	0.0764
P1 (3.24)	15.40	0.29	54.3	0.764	11.89	0.0627
P1 (5.34)	24.10	0.26	44.1	0.800	18.17	0.0705
P1 (6.90)	8.45	0.28	87.8	0.734	6.73	0.0822
P1 (9.09)	16.80	0.28	45.1	0.774	13.84	0.0658

#### 3.3.3 Effect of scan rate on PCV formation

**Figure 5:**
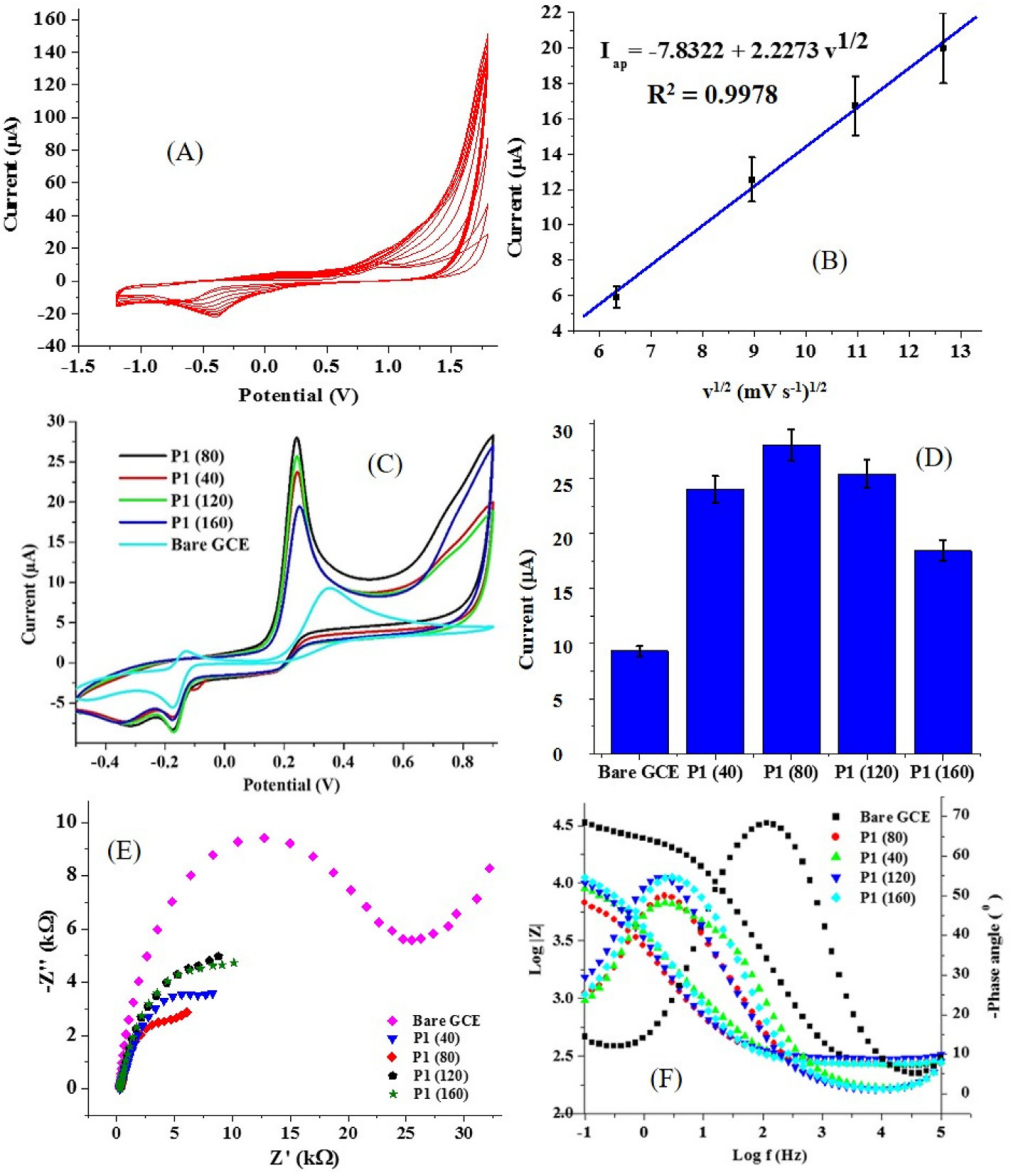
(A) Cyclic voltammogram of CRV polymerization at 40 mV s^−1^ CRV for P1 preparation, (B) Chart showing the current response of CRV (first scan) electropolymerized on GCE at various scan rates, and (C) Cyclic voltammogram showing current response of the modified electrodes P1 (40), P1 (80), P1 (120) and P1 (160) to AD oxidation at a scan rate of 25 mV s^−1^ in PBS of pH 7. (D) Chart showing the current response of the bare and modified electrodes to AD oxidation. (E) Nyquist and (F) Bode and phase angle diagram of electrodes in 0.4 mM AD at a pH of 7.

The effect of scan rate on the formation of PCV was also investigated over a scan rate range of 40–160 mV s^−1^. The electrodes were fabricated using the same polymerization conditions as P1 (as explained in section 3.1) except for changes in scan rates. The electrodes fabricated at scan rates of 40, 80, 120 and 160 mV s^−1^ were tagged P1 (40), P1 (80), P1 (120) and P1 (160), respectively. Figure 5A shows the CV for PCV formation at a scan rate of 40 mV s^−1^. This CV is similar to that of PCV formed at 80 mV s^−1^ (Figure 4A), except for the difference in the peak current recorded at the first scan. The variation of this current response with scan rate can be seen in Figure 5 B. The linear relationship between CRV oxidation current (I_ap_) (for the first scan at each scan rate) and the square root of the scan rate (v^1/2^) suggests a diffusion-controlled process [30] (Figure 5B).

In a previous report on CRV electropolymerization, it was established that a lower scan rate extends the polymerization time frame and consequently increases the thickness of the polymer. In comparison, the converse occurs at a higher scan rate [11]. The results obtained in the current study validate this assertion because the best AD oxidation current response was obtained with an electrode polymerized at an intermediate scan rate of 80 mV s^−1^ (P1 (80)) (Figure 5C & D). The presumably high thickness of the PCV on P1 (40) could be ascribed to the lower AD oxidation current response obtained at the PGCE electropolymerized at 40 mV s^−1^, which hampers electron transfer across the P1 (40) surface. At PGCE prepared at scan rates higher than 80 mV s^−1^, a lower AD oxidation current response was obtained, suggesting that the polymer films of such electrodes are probably not appropriate to give the optimum catalytic effect on AD oxidation.

The EIS data obtained at varying scan rates for bare GCE and the modified electrodes were fitted with equivalent circuits B and A (Figure 3), respectively. The Nyquist and Bode plots of the EIS data obtained at varying scan rates (Figure 5E) show that the lowest R_ct_ and impedance modulus at the lowest frequency (|Z| _at 0.1 Hz_) (Figure 5F) was recorded using P1 (80) while the highest values of these quantities were obtained using the bare electrode. Also, the magnitude of the constant phase element (Y_o_) at the electrode fabricated at 80 mV s^-1^ (P1 (80)) was the highest compared to every other electrode (Table 3).

**Table 3:** EIS parameter of bare GCE and GCE modified at various scan rates.

Electrode	R_ct_ (kΩ)	R_s_ (kΩ)	Y_o_ (μΩ^−1^s^n^)	n	|Z| _at 0.1 Hz_ (kΩ)	*X*^*2*^
Bare GCE	22.40	0.26	1.30	0.893	33.26	0.0764
P1 (40)	10.34	0.27	75.2	0.693	7.87	0.0816
P1 (80)	8.45	0.28	87.8	0.734	6.73	0.0822
P1 (120)	13.70	0.31	71.9	0.772	10.09	0.1039
P1 (160)	13.40	0.27	53.6	0.777	10.87	0.0658

As suspected earlier, the better performance of this electrode (P1 (6.9)) could be because the optimum thickness of the PCV coating was achieved at 80 mV s^−1^. This suspicion could be confirmed by the value of n and Y_o_ from the fitting of the EIS data of all the fabricated sensors. Considering the polymer film at the surface of the electrode as a perfect coating and a dielectric between a double layer comprising of the PBS-adrenaline mixture and the surface of the bare GCE, CPE exponent (n) gives an insight into the deviation of the coated GCE from absolute capacitive behaviour.

(1) $$Z_{CPE}=1/\left(Y_{o}{\left(j\omega \right)}^{n}\right)$$

(2) $$C_{dl}=\frac{\varepsilon \varepsilon_{o}A}{d}$$

The exponent, n, as seen in Eq. (1) shows a better deviation of the modified electrode from an ideal capacitive material as it moves further away from unity [31, 32]. Compared to every other electrode, P1 (80) gave the lowest value of n. The value of Y_o_ which can otherwise stand as the pseudo coating capacitance [33, 34] is also highest at P1 (80), indicating that the dielectric polymer film probably had the lowest thickness of all the electrodes [35]. This suggestion can be linked to the relationship between the thickness of a dielectric being in inverse proportion to Y_o_ (Eq. (2)). This conclusion can further be substantiated by the fact that the electrode tagged P1 (160) made with the highest scan rate had the lowest Y_o_ value and offered the lowest AD oxidation current response. Also, the phase angle diagram suggests that the PCV film of all the modified electrodes is a perfect homogeneous film because only one time-constant was observed in all of the modified electrodes. This is because films composed of two different layers have phase angle diagrams showing two peaks, suggesting two-time constants [32]. The phase angle of all electrodes falling below −90 ° is also an indication of deviation from an ideal capacitive behaviour and association with a resistive behaviour [35, 36, 37].

The Bode plot revealed that the lowest value of the modulus of impedance at low frequency was obtained at P1 (80), thus confirming the Nyquist plot data. Also, the Bode plot showed that the electrodes all have a resistive behaviour at a very high frequency while a capacitive behaviour could hardly be found at a low frequency [35]. The dominance of this resistive behaviour indicates the conductive nature of all the electrodes since such a material would be expected to allow the passage of electric current with low resistance compared to a material with a capacitive behaviour. This is consistent with the CV data (Figure 5C). Notably, the poorest current response was obtained at P1 (160). Clearly, the Nyquist and the Bode plot showed that the R_ct_ and the value of |Z| _at 0.1 Hz_ at higher scan rates (120 and 160 mV s^−1^) are very close, thus confirming the adverse effect of higher scan rate in preparing polymer films with optimum conductivity towards AD.

**Scheme 2:**
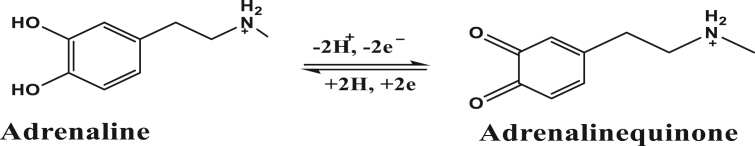
Redox reaction of adrenaline.

It is equally important to note that the CV obtained over 12 scans for the PCV formed at various scan rates proved that very stable PCV films were formed after the first three scans during P1 (80) formation. In contrast, other electrodes showed a steady drop in oxidation current till the very last scan (Figure 2A). This suggests that the P1 (80) could have been an electrode with the most stable PCV film. The modified electrode obtained at a pH of 6.9 and scan rate of 80 mV s^−1^ using CRV concentration of 0.2 mM was tagged PGCE and subsequently used as the working electrode in other voltammetric studies. The detection of AD involves the reversible loss of two protons and two electrons by AD to form adrenalinequinone (Scheme 2). The oxidation reaction (formation of adrenalinequinone) led to the emergence of the prominent anodic peak in CV while the reverse process (reduction of adrenalinequinone to AD) gave the prominent cathodic peak.

The EIS data of the bare electrode showed that it exhibited greater capacitive behaviour than every other electrode, confirming the conductive nature of the PCV films in all modified electrodes. This can be seen from the phase angle diagram of the bare GCE in AD where the smallest deviation from a phase angle of −90 ° was observed [32,35]. The low Y_o_ value recorded at the bare electrode showed that it is a completely different material with an entirely different dielectric when immersed in AD. The EIS and CV characterization showed that the best conductive material for AD detection at the PCV-modified GCE is P1. This electrode was named PGCE and used for further studies.

## 4 Electroanalysis of AD at PGCE

### 4.1 Effect of scan rate on AD oxidation at PGCE

**Figure 6:**
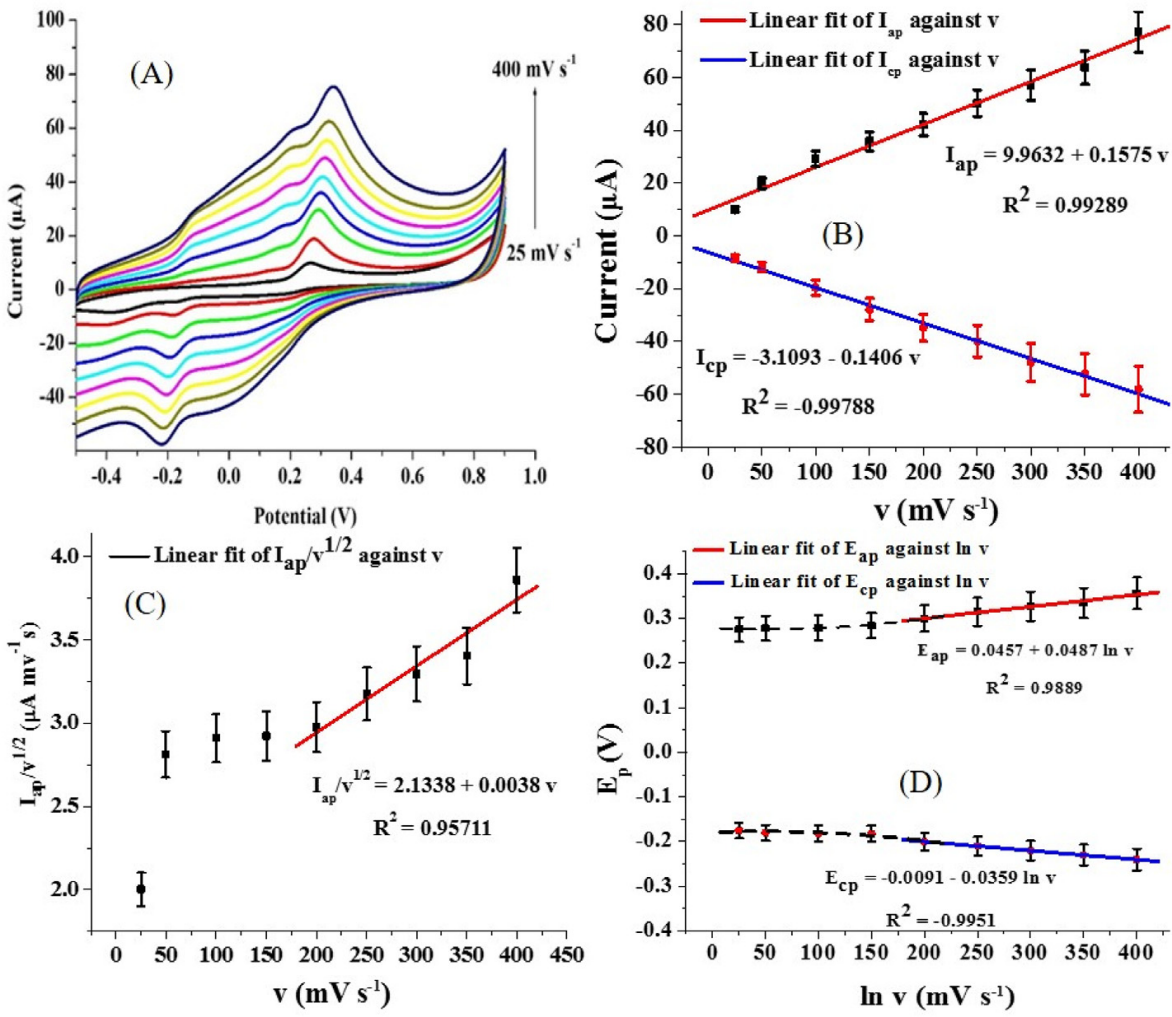
(A) Cyclic voltammogram of 0.2 mM AD current response at PGCE at a scan rate range of 25–400 mV s^−1^ (pH 7), (B) Chart of the relationship between I_p_ and v, (C) Plot of I_ap_/v^1/2^ versus v, and (D) Plot of E_p_ against ln v.

The scan rate studies showed that the current response of the anodic reaction increased as the scan rate increased with a shift of the anodic peak potential to the right (positive end). The cathodic reaction showed that the reduction current increased (negatively) as the scan rate increased with a shift of the cathodic peak potential to the left (Figure 6 A). This suggests that the redox reaction at the electrode is a quasi-reversible one. The scan rate (v) varies linearly with the anodic and the cathodic peak currents (I_ap_ and I_cp_) with R^2^ values of 0.993 and 0.988, respectively (Eqs. (3) and (4), Figure 6 B). This indicates that the redox process at the surface of PGCE is probably diffusion-controlled.

(3) $$\;I_{ap}=9.9632+0.1575\;v\;\left(R^{2}=0.9929\right)$$

(4) $$I_{cp}=-3.1093-0.1406\;v\;\left(R^{2}=0.9979\right)$$

(5) Iapv1/2=2.1338+0.0038v(R2=0.9571)

To further probe the nature of the interaction between AD and PGCE, the possible rate-limiting step of the redox process was investigated through the plot of I/v^1/2^ against the scan rate (v). As earlier reported, a linear relationship between these quantities suggests the transfer of charges from the adsorbed AD on PGCE is the dominant reaction mechanism, while the independence of I_ap_/v^1/2^ suggests that the AD molecules diffuse to the PGCE surface prior to electron transfer [38]. After plotting I_ap_/v^1/2^ against v, a linear relationship was only observed after 200 mV s^−1^ while the total independence of I_ap_/v^1/2^ was inherent at a lower scan rate (Figure 6 (C)).

This confirms the mixed surface phenomenon and, the diffusive mechanism earlier suspected. Eq. (5) shows the mathematical relationship between I_ap_/v^1/2^ and v. Noteworthy, the increase in the potential with an increase in the scan rate is almost unnoticeable at scan rates lower than 200 mV s^−1^ (Figure 6 D). According to the Laviron model, such behaviour can be described by the linear equation obtained from a plot of the natural logarithm of v (ln v) and the peak potential (E_p_). The slope (Tafel slope) of this curve is −RT/αnF and RT/RT/(1 − α)nF for the cathodic and the anodic reaction (Eqs. (6) and (7)), respectively [38]. R, T, α, n and F represent the molar gas constant (J.mol.K^−1^), temperature (T), charge transfer coefficient, and Faraday’s constant (C.mol^−1^). The Tafel values for both the anodic (0.224 V dec^−1^) and cathodic reaction (0.165 V dec^−1^) were higher than a value of 0.118 V dec^−1^ reported for a one-electron process [39]. These Tafel values are similar to those obtained for AD detection at MWCNTs-PANI-RuO_2_ modified Au electrode [40]. This could be because of surface phenomena such as adsorption of AD or its quinone form onto the surface of the electrode.

(6) $$E_{cp}=-0.0091-0.0359\;\ln\;v\;\left(R^{2}=0.9951\right)$$

(7) $$\;E_{ap}=0.0457+0.0487\;\ln\;v\;\left(R^{2}=0.9889\right)$$

The value of α obtained from the mathematical computation of the Tafel slope of the anodic and the cathodic reactions was 0.58. This value of α is similar to the value reported for some chemically modified electrodes targeted towards biomolecule detection [41], thus suggesting the successful electrocatalysis of AD oxidation at PGCE. Given that the number of electrons transferred in the redox reaction of AD at PGCE is 2 (Scheme 2), the charge transfer rate constant (k_s_) was calculated as 2.76 × 10^−5^ s^−1^ using Eq. (8).

(8) $$logk_{s}=\alpha \;\log\left(1-\alpha \right)+\left(1-\alpha \right)log\alpha -\log\left(\frac{RT}{nFv}\right)-\frac{nF\Delta Ep\left(1-\alpha \right)}{2.3RT}$$

### 4.2 Effect of pH on AD oxidation at PGCE

**Figure 7:**
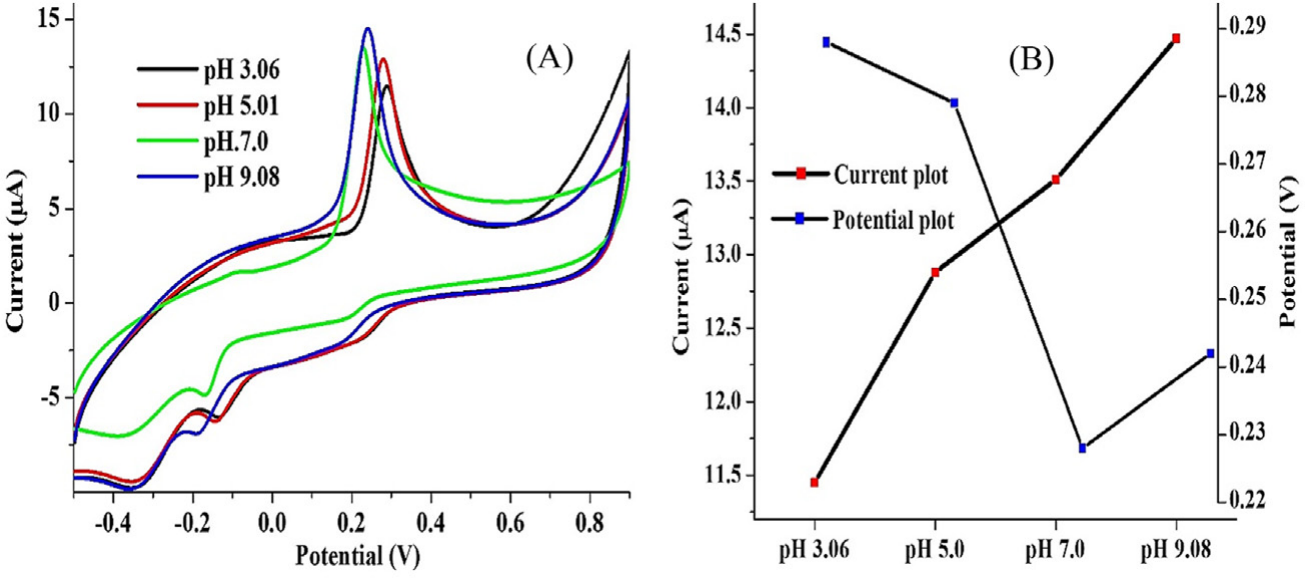
(A) Cyclic voltammogram of 0.2 mM AD oxidation at PGCE at various electrolyte pH (scan rate: 25 mV s^−1^) and (B) graphical representation of current response and peak potential at various pH.

The pH dependence of the electrocatalytic activity of PGCE on AD was investigated using 0.2 mM AD at a scan rate of 25 mV s^−1^ over a pH range of 3.06–9.08. The best AD current response was obtained at a pH of 9.08, while the closest current response to that was obtained at a pH of 7.0 (Figure 7A). The lowest oxidation peak current was obtained at the lowest and the most acidic pH (3.06). This suggests that the AD oxidation at PGCE is best achieved at high pH while lower pH retards the electron transfer process. Generally, the AD oxidation peak current increased with an increase in the pH, while the converse is true for the pH-peak potential curve except for an increase observed at pH 9.08 (Figure 7B). The optimum pH recorded here is higher than a pH of 7–8, often reported for AD detection and a pH of 5 specifically reported for AD detection using a Co-phthalocyanine/single-walled carbon nanotube modified electrode [42]. The pKa of the hydroxyl groups of AD has been reported to be within the range of 8.7–12 while that of the amino functional group was reported as 9.9 [42]. This implies that the AD molecule acts as a weak acid with partial ionization in an aqueous medium. At a very high pH, the hydroxyl group of AD deprotonates, giving a negatively charged –OH which in turn is electrostatically attracted to the positive end of the PCV. AD exists in the human system at a physiological pH of ∼7, therefore, we stick to a pH of 7 for subsequent electroanalysis of AD [43, 44].

### 4.3 Effect of concentration on AD oxidation

**Figure 8:**
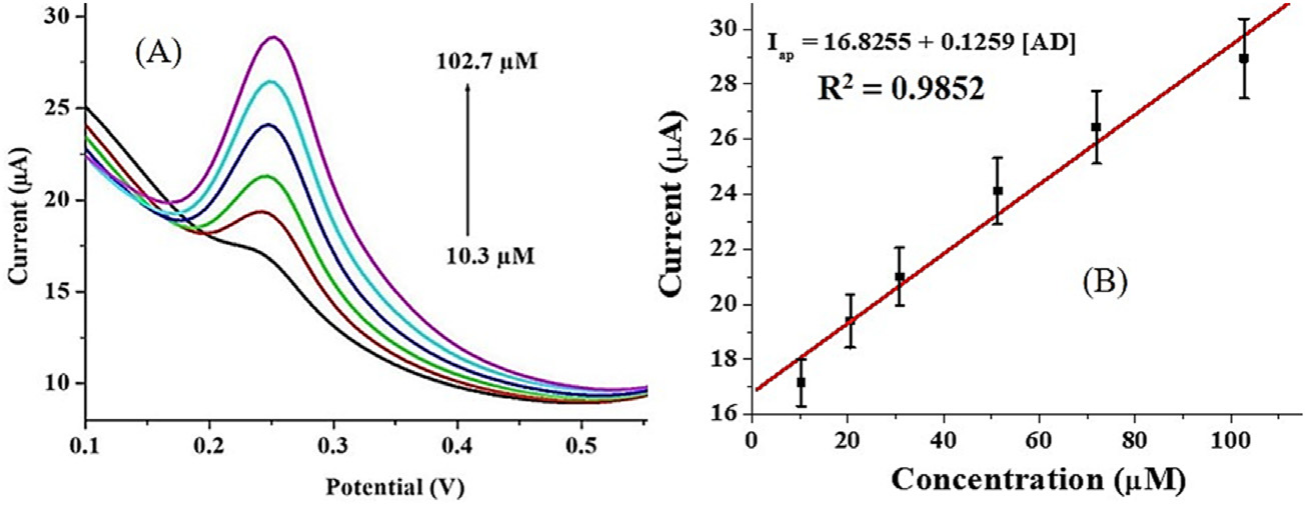
(A) Cyclic voltammogram of varying AD concentration (10.3–102.7 μM) at PGCE (pH 7, scan rate 25 mV s^−1^) and (B) Chart representing I_ap_ against AD concentration.

The effect of concentration on the electrocatalytic oxidation of AD at PGCE was investigated using 10.3–102.7 μM AD solution in a PBS buffer of pH 7.0 at a scan rate of 25 mV s^−1^. Adrenaline peak was observed at a peak potential of 0.25 V (Figure 8A). As seen in the square wave voltammogram, the oxidation peak currents increased as the concentration of AD increased with a linear relationship I_ap_ = 16.8255 + 0.1259 [AD]. The high value of the correlation coefficient (R^2^ = 0.9852) suggests a good fit between the current response and the AD concentration (Figure 8B). From this linear relationship, the limit of detection (LOD) of PGCE was calculated as 2.86 μM. The LOD was calculated using the relationship LOD = 3ρ/m, where ρ is the standard deviation of the blank (n = 20) and m is the slope of the calibration curve [24]. Figure S4 shows the CV for the replicate blank determinations. The sensor gave this linear relationship over a dynamic range (LDR) of 10.3–102.7 μM. The LOD and LDR obtained in this study are comparable with that of a wide range of sensors in literature (Table 4). Specifically, the proposed sensor gave a lower LOD than some existing electrochemical AD sensors [45, 46]. This implies that it could be accurately applied for AD detection at the micromolar level over a wide LDR.

**Table 4:** Comparison of the performance of PGCE and previous adrenaline sensors.

Electrode	Method	LOD (μM)	LDR (μM)	References
Au nanoporous film/Au	CV	19	50–1000	[45]
Au–Ag electrode	LSV	14.7	25–300	[46]
Au–Ag electrode	DPV	5.05	25–700	[46]
GPE/nps-CuTe	DPV	0.018	5–60	[48]
CRGO/GCE	DPV	1.6	10–300	[49]
SWCNTs/SME	DPV	2.0	2–100	[50]
PGCE	SWV	2.86	10.3–102.7	This work

Au nanoporous film/Au – Nanoporous gold film modified gold electrode; Au–Ag – Nanoporous gold-silver film modified gold electrode; GPE/npa-CuTe – Copper Telluride nanoparticles modified graphite paste electrode; CRGO/GCE – GCE modified by chemically reduced graphene oxide; SWCNTs/SME – Stainless steel micro electrode modified functionalized single-walled carbon nanotube.

Moreover, the proposed sensor possesses a high level of simplicity and ease of fabrication which might be difficult to match by some existing AD sensors fabricated through the more laborious drop-casting technique using modifiers composed of two or more materials [14,47].

### 4.4 Interference studies

**Figure 9:**
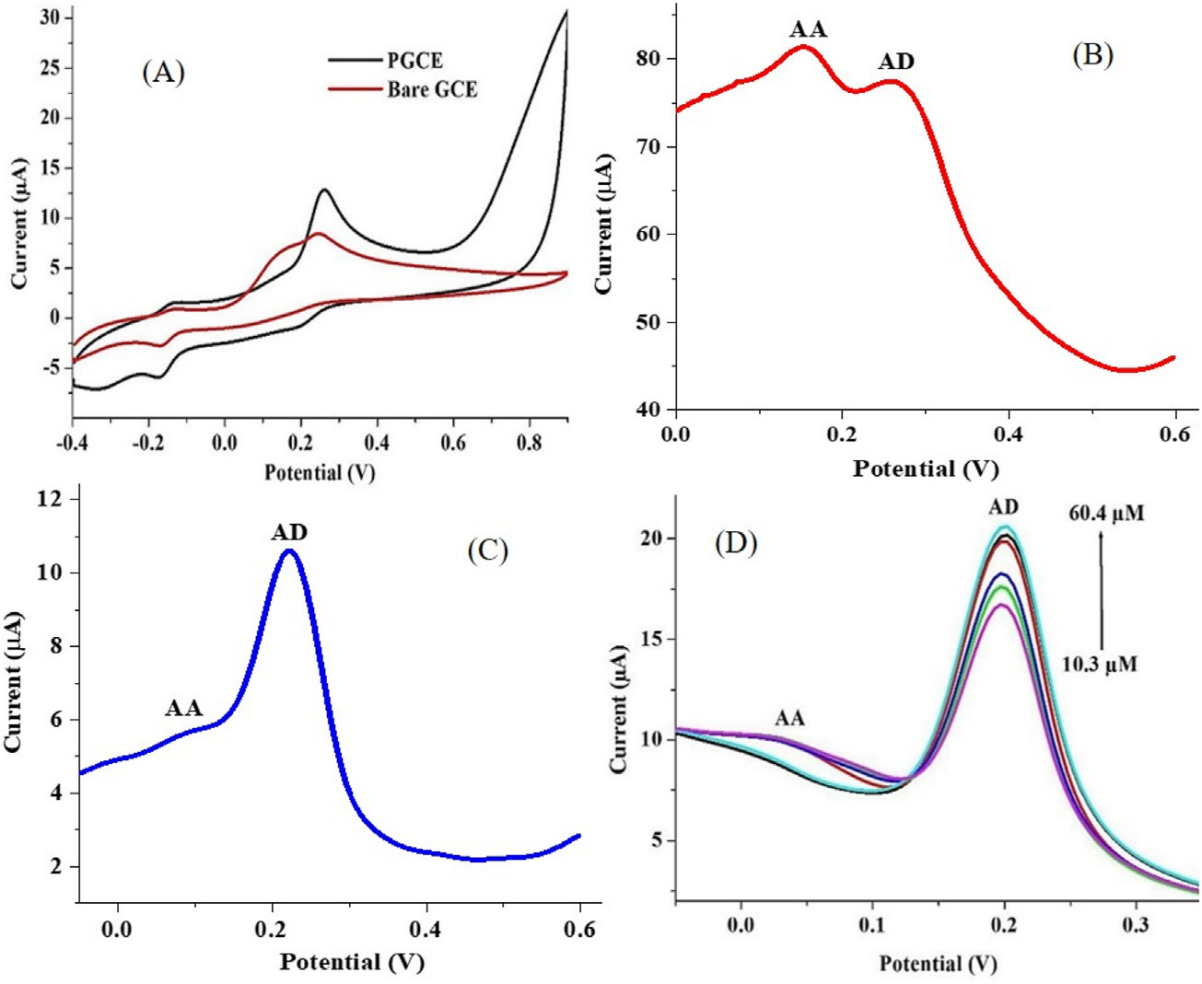
(A) Cyclic voltammogram of bare GCE and PGCE (B) square wave voltammogram and (C) differential pulse voltammogram of PGCE in 0.2 mM AD and 1 mM AA. (D) Differential pulse voltammogram of AD concentration increase (10.3–60.4 μM) with constant AA concentration (1 mM) at PGCE (pH 7, scan rate 25 mV s^−1^).

Adrenaline exists in the human system alongside some other biomolecules whose oxidation potential is similar to that of AD. These biomolecules are often present at a much higher concentration than AD, such that the electrochemical detection of AD in the presence of conventional electrodes is difficult to achieve. Ascorbic acid is one of the biomolecules found in physiological fluids with AD [43]. In this study, we undertook the determination of AD in the presence of AA using differential pulse voltammetry (DPV) at a pH of 7.0. AA showed an oxidation peak at 0.15 V while the AD peak was observed at 0.25 V (Figure 9C). Using cyclic voltammetry (CV), the peaks were almost indistinguishable (at both bare GCE and PGCE) (Figure 9A), while well-resolved peaks were obtained with the use of SWV and DPV (Figure 9B & C). This confirms the renowned suppression of charging current by the SWV and DPV. However, there is a discrepancy in the response of PGCE using these two techniques. The AA-AD peak separation using DPV was 189 mV (Figure 9C), while a peak separation of 126 mV was recorded with the SWV technique (Figure 9B).

This explains why the DPV was selected for the interference studies. Interestingly, at lower AD concentrations, the AA-AD peak separation slightly improved. The DPV current response obtained for AD oxidation increased while the AA concentration (1 mM) remained constant, suggesting that PGCE is capable of AD detection in the presence of high AA concentration (Figure 9D). Also, there was a drop in the peak for AA at higher AD concentrations. This could be attributed to the more favoured transportation of the protonated AD (cationic) molecule towards the more electronegative amino nitrogen atoms on PCV (Scheme 1) with the increase in AD concentration. On the other hand, the anionic AA suffered repulsion by the polymeric PCV film.

### 4.5 Real sample analysis

The real sample analysis was done by analysing adrenaline in adrenaline injection through the standard addition method [51]. The mean percentage recovery of adrenaline from the adrenaline injection solution is 98.9% (n = 3). The percentage relative standard deviation (% RSD) of the triplicate measurement depicted in Table 5 is 7.4%.

**Table 5:** Real sample analysis data (n = 3).

Sample	Amount added (μM)	Amount found (μM)	Recovery (%)
Adrenaline injection	40.0	41.6	103.0
	40.0	36.2	90.5
	40.0	41.3	103.3

### 4.6 Stability and reproducibility

**Figure 10:**
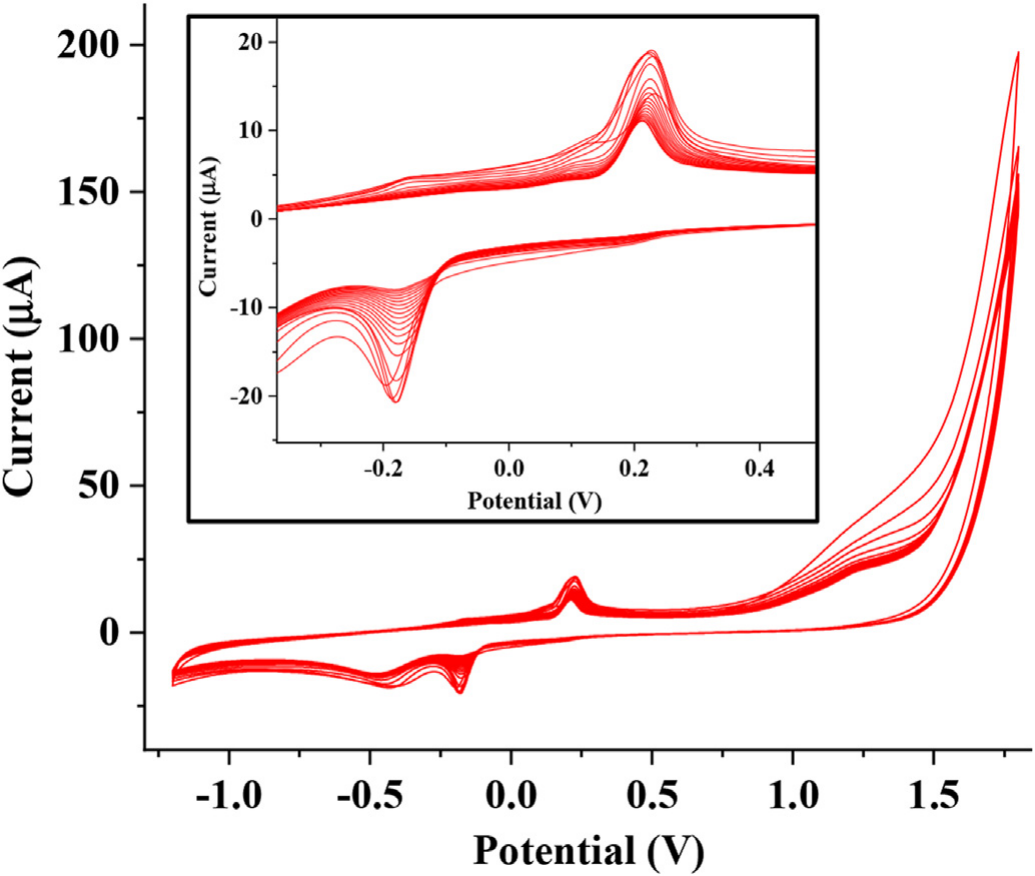
Cyclic voltammogram of PGCE in 0.2 mM AD over 20 scans (pH 7, scan rate 25 mV s^−1^) (inset: the zoomed redox peaks).

The stability of the electrode was investigated by acquiring 20 CV scans over a potential range of −1.2 to 1.8 V at a pH of 7.0 and a scan rate of 25 mV s^−1^ in the presence of AD (Figure 10). The current dropped from a maximum value of 19.06 μA at the first scan to 11.14 μA at the end of the 20th scan. This implies that PGCE lost about 41.6 % of its initial current response, suggesting that the electrode is relatively stable compared to some other electrodes that suffered far greater current response loss after several CV scans [52]. Also, the reproducibility of the electrode was investigated by modifying three different electrodes with PCV and subsequently applying them for AD detection. The PCV-modified electrodes gave CV current response to AD oxidation with a % RSD of 1.8%. This suggests that PGCE fabrication for AD detection is reproducible with a low error margin.

## 5 Conclusion

Electrochemical AD detection at an optimized PCV film-modified GCE (PGCE) was attempted for the first time in this study. The effect of pH, scan rate, and monomer concentration on the thickness and conductivity of the polymeric film was investigated using CV and EIS to establish the optimum conditions for PGCE fabrication. The electrode offered good stability, a wide linear range and a relatively low detection limit. The sensor’s simplicity, stability, reproducibility, selectivity, and sensitivity are strong indications that PGCE is a reliable tool for electrochemical AD detection. The high % recovery of AD from adrenaline injection (98.9%) confirms the suitability of PGCE for point-of-care AD detection at the micromolar level. This study also proved that high scan rates and CRV concentrations could be detrimental to the formation of very stable PCV films.

## Declarations

### Author contribution statement

Saheed E. Elugoke: Performed the experiments; Analyzed and interpreted the data; Wrote the paper.

Omolola E. Fayemi: Conceived and designed the experiments; Analyzed and interpreted the data; Contributed reagents, materials, analysis tools or data; Wrote the paper.

Abolanle S. Adekunle, El-Sayed M. Sherif: Analyzed and interpreted the data; Wrote the paper.

Eno E. Ebenso: Conceived and designed the experiments; Analyzed and interpreted the data; Wrote the paper.

### Funding statement

This work was supported by King Saud University (RSP-2021/33).

### Data availability statement

Data will be made available on request.

### Declaration of interests statement

The authors declare no conflict of interest.

### Additional information

No additional information is available for this paper.

## Appendix A Supplementary data

Supplementary Material _spl_Adrenaline_spl_ Heliyon ACCEPTED_V3 Supplementary Material _spl_Adrenaline_spl_ Heliyon ACCEPTED_V3
