# Supplementary material for: Electrochemical sensor for the detection of adrenaline at poly(crystal violet) modified electrode: optimization and voltammetric studies
Source: Heliyon. 2022 Oct 4;8(10):e10835. doi: 10.1016/j.heliyon.2022.e10835 (PMC9573894; doi:10.1016/j.heliyon.2022.e10835)
Supplement: Supplementary Material _spl_Adrenaline_spl_ Heliyon ACCEPTED_V3 [file mmc1.docx]

**Supplementary Material**

**Effect of concentration**

The synthesis of PCV was done using 0.1 mM (P0), 0.2 mM (P1), 0.4 mM (P2) and 0.8 mM (P3) of CRV in the presence of sodium nitrate in PBS. The electrodeposition of PCV on bare GCE was done at 80 mV s^-1^ scan rate over a potential window of -1.2 to 1.8 V. Figure S1 shows the cyclic voltammogram generated after the synthesis of the respective PVC.

**Figure S1: Cyclic voltammogram for the synthesis of P0, P1, P2 and P3**

**Effect of pH**

The preparation of PCV at pH 3.24, 5.34, 6.90 and 9.09 was carried out at 80 mV s^-1^ using the optimum CRV concentration (0.2 mM in the presence of sodium nitrate to obtain modified electrodes tagged P1 (3.24), P1 (5.34), P1 (6.90) and P1 (9.09), respectively . Figure S2 shows the CV generated PCV electrodeposition on bare GCE.

**Figure S2: Cyclic voltammogram for the synthesis of P1 (3.24), P1 (5.34), P1 (6.90) and P1 (9.09)**

**Effect of scan rate**

The electrodeposition of PCV on bare GCE at 40, 80, 120 and 160 mV s^-1^ at pH 6.9 using 0.2 mM CRV was done to obtain electrodes tagged P1 (40), P1 (80), P1 (120) and P1 (160), respectively. Figure S3 depict the CV generated after the fabrication of these electrodes.

**Figure S3: CV for the synthesis of P1 (40), P1 (80), P1 (120) and P1 (160)**

**Concentration studies**

**Calibration curve blank determination**

Figure S4 shows the 20 CV scans of the blank used for the calculation of the detection limit in section 4.3

**Figure S4: Cyclic voltammogram of 20 blank determinations using 0.1 M PBS (pH 7)**
